# Impact of Preoperative Hypocalcemia on Complications Following Total Shoulder Arthroplasty: A Propensity Score Matching Study

**DOI:** 10.7759/cureus.104632

**Published:** 2026-03-03

**Authors:** Andrew Moyal, Luc Fortier, Nathan Cuttica, Jeremy M Adelstein, Robert J Burkhart, Rayyan Abid, Michael J Salata, Robert J Gillespie, James E Voos, Jacob Calcei

**Affiliations:** 1 Orthopaedic Surgery, University Hospitals Drusinsky Sports Medicine Institute, Cleveland, USA; 2 Orthopaedics, University Hospitals Drusinsky Sports Medicine Institute, Cleveland, USA

**Keywords:** arthroplasty, bone mineral density, calcium score, hypocalcemia, total shoulder arthroplasty

## Abstract

Introduction

Hypocalcemia is a common metabolic deficiency, notably impacting patients above 65 years of age. With the increase in volume of total shoulder arthroplasty (TSA), it is important to understand how hypocalcemia may impact complication rates. The aim of this study was to analyze the rates of complication following TSA in hypocalcemic patients, serum calcium < 8.5 mg/dL, compared to those with normocalcemia, serum calcium >8.5 mg/dL.

Materials and methods

Utilizing TriNetX, healthcare organizations within the United States were queried to create two cohorts consisting of patients undergoing TSA (Current Procedural Terminology (CPT): 23472) with hypocalcemia within one month of surgery (hypocalcemia cohort) and normocalcemia within one month of surgery (normocalcemia cohort). Primary outcomes included the odds of emergency department (ED) visit, inpatient admission, wound dehiscence, intraoperative fracture, postoperative hematoma or seroma, prosthetic joint infection, joint loosening, periprosthetic shoulder fracture, and reoperation. Outcomes were stratified by timeframe and were analyzed before and after propensity score matching.

Results

Prior to propensity score matching, patients with hypocalcemia had an increased rate of intraoperative fracture (odds ratio (OR) 2.9, p<0.01) and postoperative hematoma or seroma (OR 2.6, p < 0.01) at seven days postoperatively. At the two-month timeframe, hypocalcemic patients demonstrated increased odds of prosthetic joint infection (OR 1.7, p<0.05), joint loosening (OR 1.8, p<0.05), periprosthetic shoulder fracture (OR 1.8, p<0.05), and reoperation (OR 1.6, p<0.05). Results at two-years demonstrated similar findings. After propensity score matching, minimal clinical differences other than ED visits and joint loosening were seen between the cohorts.

Conclusions

Prior to propensity score matching, hypocalcemic patients demonstrated increased odds of implant-related complications within seven days, two months, and two years after TSA. However, after propensity score matching for patient demographics and comorbidities, only joint loosening and ED visits demonstrated a statistically significant difference between cohorts. Our results suggest that chronic hypocalcemia in patients with other comorbidities can potentially serve as a proxy for the odds of postoperative complications. Additionally, our results suggest that correcting hypocalcemia prior to TSA can potentially improve postoperative outcomes.

## Introduction

Background

Hypocalcemia is defined by a serum calcium concentration less than 8.5 mg/dL and is a common mineral deficiency occurring in 28% of patients and 61% of patients over 65 years of age [1]. The origin of hypocalcemia is commonly linked to vitamin D deficiency, renal disease, gastrointestinal disorders affecting calcium absorption, and hypoparathyroidism [2]. Roughly 99% of the body’s calcium is found in the bone and teeth, and 40% of bone weight can be attributed to calcium, primarily in the form of hydroxyapatite. Consequently, chronic hypocalcemia has been associated with significant bone pathologies that impact skeletal fragility and bone density, resulting in an increased risk of fracture [3].

Biomechanical studies have demonstrated the effect of low bone density and the association with difficulty in the anchoring of implants and the strength of internal fixation [4]. Yet, clinical studies have failed to produce similar results when the data are pooled, primarily due to the lack of accurate osteoporosis evaluation and heterogeneous inclusion criteria [5]. Given the challenges with obtaining site-specific bone mineral density (BMD) measurements and the questionable validity of the Singh radiographic index, serum calcium may be a simple and effective proxy to measure preoperatively to help identify patients at risk [6,7].

Rationale

Given the rapid increase in total shoulder arthroplasty (TSA) over the last two decades and increased ease of teasing preoperative calcium levels, the purpose of this analysis is to investigate hypocalcemia as a factor for increased postoperative complications in TSA. [8]. Specifically, the authors sought to answer two main questions: (i) How does TSA in patients with a serum calcium of <8.5 mg/dL impact complication rates compared to patients with normal serum calcium levels before accounting for comorbidities of hypocalcemia via propensity score matching? (ii) How do complication rates in hypocalcemic patients compare to normocalcemic patients after propensity matching for comorbidities contributing to hypocalcemia?

## Materials and methods

Study design and setting

This study applied the Strengthening the Reporting of Observational Studies in Epidemiology (STROBE) guidelines to the TriNetX analytics platform to create a nationwide analysis that represents patients from the United States [9]. TriNetX is a platform that contains over 110 million patients from greater than 60 healthcare organizations across the United States. Data in TriNetX is directly derived from the Current Procedural Terminology (CPT) and International Classification of Diseases (ICD) codes of each contributing health care organization. Due to the nature of the de-identified data and blurring of single-digit outcomes, this study has been deemed non-human research by the Case Western Reserve University Institutional Review Board (IRB) and, thus, did not require IRB approval.

Participants

TriNetX was queried on June 11, 2024 to establish a cohort of patients undergoing either anatomic or reverse TSA (CPT: 23472) with a serum calcium <8.5 mg/dL within one month prior to surgery (hypocalcemia cohort) and a cohort of patients with a serum calcium 8.5-10.5 mg/dL within one month prior to surgery (normocalcemia cohort). Groups were created using both CPT codes and laboratory data derived from the electronic health record of each institution participating in TriNetX. Data was selected up to June 11, 2022, to enable at least a two-year follow-up in the patients included.

Study outcomes and process

Primary outcomes of interest included the odds of emergency department (ED) visit, inpatient admission, wound dehiscence, intraoperative fracture, postoperative hematoma or seroma, prosthetic joint infection, joint loosening, periprosthetic shoulder fracture, and reoperation. Forms of reoperation included arthrotomy, removal of prosthesis, revision TSA, arthroscopic synovectomy, and insertion of a drug-delivery implant. Outcomes of interest and associated CPT and ICD codes are shown in the Appendices. Additionally, patient demographics, including age, race, and sex, were recorded.

Outcomes collected were stratified into three separate timepoints: immediate intraoperative and postoperative outcomes from 0-7 days after surgery to evaluate intraoperative fracture or operative complications that could have resulted in inpatient hospitalization, acute postoperative outcomes seven days to two months, and two months to two years postoperative to adequately capture late outcomes, such as reoperation and periprosthetic joint infection [10-12].

Outcomes were analyzed for the two cohorts prior to and after propensity score matching at each timepoint. Post-match analysis was completed to assess the impact of underlying demographics and medical comorbidities known to be risk factors for hypocalcemia.

Statistical analysis

All statistical analysis was completed with the built-in tools of the TriNetX platform. Frequencies were assessed via chi-square analysis. Mean and standard deviations (SD) for continuous variables were assessed with Mann-Whitney U testing. Outputs provided the odds ratio (OR) and 95% confidence interval (CI) for each outcome assessed. Statistical significance was set at p < 0.05. Utilizing a greedy-nearest-neighbor matching algorithm, a one-to-one propensity score matched the control and hypocalcemic cohorts based on demographics and known risk factors for hypocalcemia. Propensity score matching was performed for age, race, sex, osteoporosis (ICD: M81), hyperparathyroidism (E20), and chronic kidney disease (N18) as these are known risk factors for hypocalcemia or worse outcomes after TSA [2,13]. The caliper distance was set as equal to 0.1 pooled standard deviation of the logit of the propensity score.

## Results

**Figure 1:**
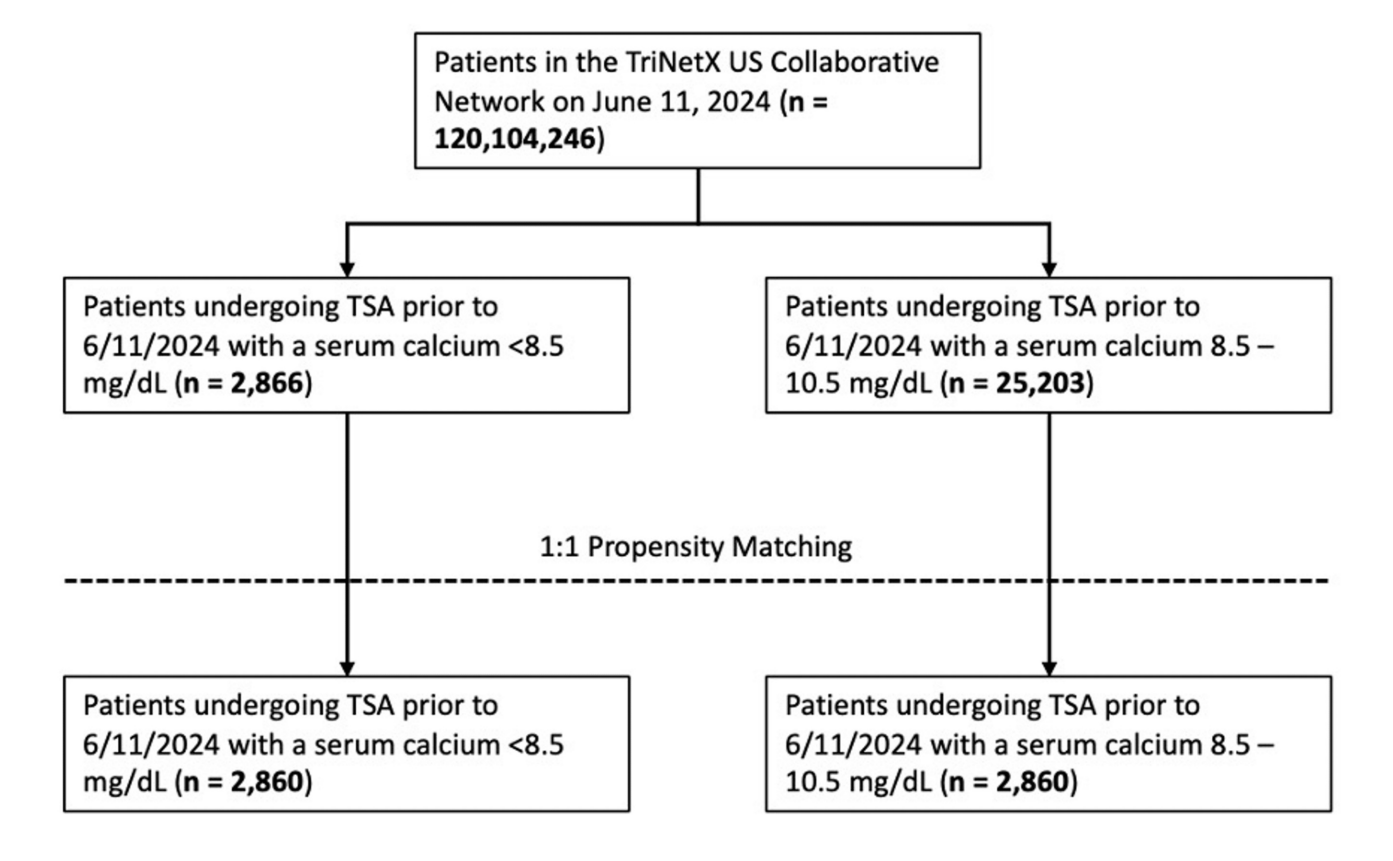
Creation of cohorts according to the STROBE guidelines STROBE: Strengthening the Reporting of Observational Studies in Epidemiology; TSA: total shoulder arthroplasty

Of the total patients in the TriNetX database, 2,866 met criteria for the hypocalcemia cohort, and 25,203 met criteria for the normocalcemia cohort. After propensity score matching, there were 2,860 participants in each cohort (Figure 1).

Utilizing the summary statistics tools within TriNetX, both cohorts were verified to have >80% follow-up at the two-year mark. The normocalcemic cohort had a 93% follow-up through two years, while the hypocalcemic cohort had 91% follow-up.

Comparison of complication rates between cohorts before propensity matching for comorbidities contributing to hypocalcemia

Without propensity score matching, 2,866 patients met criteria for hypocalcemia, and 25,203 met criteria for normal serum calcium (Table 1). The mean age was 70 and 69 years in the hypocalcemic and normocalcemic cohorts, respectively. From the time of operation (Day 0) through seven days postoperatively, hypocalcemic patients had significantly higher rates of intraoperative fracture (OR 2.9, 95%CI 1.5 - 5.5, p < 0.01) and postoperative hematoma or seroma (OR 2.6, 95%CI 1.3 - 5.1, p < 0.01) (Table 2).

**Table 1:** Demographics and comorbidities of the normocalcemia and hypocalcemia cohorts prior to propensity score matching. p < 0.05 is considered significant Ca: calcium; SD: standard deviation

Variable	Serum Ca < 8.5 mg/dL (n=2,866)	Serum Ca 8.5 – 10.5 mg/dL (n=25,203)	P-value	Std. Difference
Age (years), mean ± SD	70 ±10	69 ±20	< 0.01	0.110
Black or African American, n (%)	191 (6.7%)	1,709 (6.8%)	0.78	0.005
Male, n (%)	1,143 (39.9%)	10,472 (41.1%)	0.07	0.037
Osteoporosis without current pathological fracture, n (%)	222 (7.8%)	4,718 (4.7%)	< 0.01	0.126
Hypoparathyroidism, n (%)	10 (0.3%)	31 (0.1%)	<0.01	0.047
Chronic kidney disease (CKD), n (%)	347 (12.1%)	5,098 (6.8%)	<0.01	0.182

**Table 2:** Unmatched analysis comparing hypocalcemic and normocalcemic patients 0-7 days after total shoulder arthroplasty. *Odds ratios compare hypocalcemia to normocalcemia cohort. p < 0.05 is considered significant

Complication	Odds Ratio*	95% CI	P-value	z- statistic
Intraoperative Fracture	2.9	(1.5, 5.5)	< 0.01	3.29
Postopertive Hematoma/Seroma	2.6	(1.3, 5.1)	< 0.01	2.90

From one week to two months following TSA, hypocalcemic patients had significantly higher rates of ED visits (OR 1.5, 95%CI 1.3-1.8, p <0.01) and inpatient admissions/observation (OR 2.8, 95%CI 1.8-4.2, p <0.01). Additionally, hypocalcemic patients also demonstrated significantly higher rates of joint loosening and dislocation (OR 1.8, 95%CI 1.3-2.3, p <0.01), periprosthetic fracture (OR 1.8, 95%CI 1.1-3.0, p = 0.02), prosthetic joint infection (PJI) (OR 1.7, 95%CI 1.2-2.5, p <0.01) and reoperation (OR 1.6, 95%CI 1.2-2.1, p <0.01). Reoperation included arthrotomy, removal of prosthesis, revision TSA, arthroscopic synovectomy, and insertion of a drug-delivery implant. There was no significant difference identified in rates of wound dehiscence or periprosthetic fracture between the groups (Table 3).

**Table 3:** Unmatched analysis comparing hypocalcemic and normocalcemic patients seven days to two months after total shoulder arthroplasty. *Odds ratios compare hypocalcemia to normocalcemia cohort. p < 0.05 is considered significant

Complication	Odds Ratio*	95% CI	P-value	z- statistic
Emergency Department Visits	1.5	(1.3, 1.8)	< 0.01	5.73
Inpatient Admission/Observation	2.8	(1.8, 4.2)	< 0.01	5.09
Wound Dehiscence	1.4	(0.7, 2.8)	0.30	1.03
Prosthetic Joint Infection	1.7	(1.2, 2.5)	< 0.01	3.03
Joint Loosening and Dislocation	1.8	(1.3, 2.3)	< 0.01	4.05
Prosthetic Shoulder Fracture	1.8	(1.1, 3.0)	0.02	2.36
Reoperation	1.6	(1.2, 2.1)	< 0.01	2.96

From two months to two years postoperative, hypocalcemic patients experienced significantly more PJI (OR 1.4, 95%CI 1.1-1.8, p = 0.01), dislocation or loosening of the shoulder joint (OR 1.3, 95%CI 1.1-1.6, p = 0.01), and periprosthetic fracture of the shoulder (OR 1.6, 95%CI 1.1,2.3, p = 0.02). During this period, there was no statistical difference in rates of reoperation, with 3.7% of hypocalcemic patients and 3.6% of normocalcemic patients requiring reoperation (p = .69) (Table 4).

**Table 4:** Unmatched analysis comparing hypocalcemic and normocalcemic patients two months to two years after total shoulder arthroplasty. *Odds ratios compare hypocalcemia to normocalcemia cohort. p < 0.05 is considered significant

Complication	Odds Ratio	95% CI	P-value	z- statistic
Prosthetic Joint Infection	1.4	(1.1, 1.8)	0.01	2.70
Joint Loosening and Dislocation	1.3	(1.1, 1.6)	0.01	2.45
Periprosthetic Shoulder Fracture	1.6	(1.1, 2.3)	0.02	2.27
Reoperation	1.0	(0.9, 1.3)	0.69	0.40

Comparison of complication rates between cohorts after propensity matching for comorbidities contributing to hypocalcemia

Propensity score matching was performed to limit the potential impact of the following confounding variables: age, race, sex, and diagnoses of osteoporosis, hypoparathyroidism, and chronic kidney disease. After propensity match, 2,860 patients met the criteria for each cohort (Table 5).

**Table 5:** Demographics and comorbidities of the normocalcemia and hypocalcemia cohorts after propensity score matching. p < 0.05 is considered significant Ca: calcium; SD: standard deviation; Std: standardized

Variable	Serum Ca < 8.5 mg/dL (n=2,860)	Serum Ca 8.5 – 10.5 mg/dL (n=2,860)	P-value	Std. Difference
Age (years), mean ± SD	70 ±10	70 ±10	0.99	<0.001
Black or African American, n (%)	191 (6.7%)	191 (6.7%)	1.00	<0.001
Male, n (%)	1,143 (40.0%)	1,146 (4.0%)	0.94	0.002
Osteoporosis without current pathological fracture, n (%)	222 (7.8%)	224 (7.8%)	0.92	0.003
Hypoparathyroidism, n (%)	10 (0.3%)	10 (0.3%)	1.00	<0.001
Chronic kidney disease (CKD), n (%)	347 (12.1%	346 (12.1%)	0.97	0.001

From 0-7 days postoperatively, hypocalcemic patients did not have increased odds of intraoperative fracture (p = 0.67) and postoperative hematoma or seroma (p = 0.83) (Table 6).

**Table 6:** Matched analysis comparing hypocalcemic and normocalcemic patients 0-7 days after total shoulder arthroplasty. *Odds ratios compare hypocalcemia to normocalcemia cohort. p < 0.05 is considered significant

Complication	Odds Ratio*	95% CI	P-value	z-statistic
Intraoperative Fracture	1.2	(0.5, 2.8)	0.67	0.43
Postop Hematoma/Seroma	1.1	(0.5, 2.6)	0.83	0.22

From one week to two months following TSA, 8.0% of hypocalcemic patients required emergency medical services compared to 5.8% of the normal calcium group (OR 1.4, 95%CI 1.2-1.7, p< 0.01). The hypocalcemic cohort also experienced greater rates of postoperative joint loosening and dislocation (OR 1.8, 95%CI 1.2-2.7, p < 0.01). During this period, there were no significant differences in measured rates of inpatient admission, wound dehiscence, PJI, periprosthetic fracture of the shoulder, and reoperation (Table 7).

**Table 7:** Matched analysis comparing hypocalcemic and normocalcemic patients seven days to two months after total shoulder arthroplasty. *Odds ratios compare hypocalcemia to normocalcemia cohort. p < 0.05 is considered significant

Complication	Odds Ratio*	95% CI	P-value	z- statistic
Emergency Department Visits	1.4	(1.2, 1.7)	< 0.01	3.34
Inpatient Admission/Observation	1.7	(0.9, 3.0)	0.08	1.74
Wound Dehiscence	1.0	(0.4, 2.4)	0.99	-0.01
Prosthetic Joint Infection	1.3	(0.8, 2.1)	0.32	0.99
Joint Loosening and Dislocation	1.8	(1.2, 2.7)	0.01	2.70
Prosthetic Shoulder Fracture	1.8	(0.9, 3.9)	0.13	1.53
Reoperation	1.2	(0.8, 1.9)	0.34	0.95

From two months to two years postoperatively, the hypocalcemic and normocalcemic cohorts overall did not differ (Table 8). While rates of joint dislocation and loosening were higher in the hypocalcemic cohort (p < 0.03), the data are borderline (OR 1.4, 95%CI 1.04-1.9, p = 0.03).

**Table 8:** Matched analysis comparing hypocalcemic and normocalcemic patients two months to two years after total shoulder arthroplasty. *Odds ratios compare hypocalcemia to normocalcemia cohort. p < 0.05 is considered significant

Complication	Odds Ratio*	95% CI	P-value	z- statistic
Prosthetic Joint Infection	1.3	(0.9, 1.9)	0.13	1.50
Joint Loosening and Dislocation	1.4	(1.04, 1.9)	0.03	2.24
Periprosthetic Shoulder Fracture	1.4	(0.8, 2.3)	0.26	1.13
Reoperation	1.3	(0.9, 1.7)	0.11	1.52

## Discussion

In patients with low serum calcium at one-month post-TSA, there was an increased prevalence of intraoperative fracture (OR 2.9, p < 0.01) and acute post-operative hematoma/seroma (OR 2.6, p < 0.01). While rates of ED utilization and hospital admission/observation are higher, these may not directly reflect joint-related complications. More directly related to the implanted joint, hypocalcemic patients experienced greater prevalence of PJI (OR 1.7), joint loosening/dislocation (OR 1.8), periprosthetic shoulder fracture (OR 1.8), and reoperation (OR 1.6) at two-months (all p <0.05). At two years, hypocalcemic patients continued to experience higher rates of PJI (p < 0.01), joint loosening/dislocation (p < 0.01), and periprosthetic shoulder fracture (p = 0.02). While these findings are in line with other recent orthopedic literature, only ED visits and joint loosening and dislocation demonstrate an increased prevalence among hypocalcemic patients following propensity score matching. Regardless, past studies demonstrate an association between hypocalcemia, bone density, and orthopaedic outcomes.

Low calcium levels have been well-documented in the literature to adversely affect bone density, a critical factor in the success of orthopaedic implants such as TSA [14-17]. This is because the stability and longevity of prosthetic implants are dependent on bony ingrowth over time for secure fixation, which is contingent on adequate bone density [18]. Consequently, the results of our study demonstrating increased loosening rates over time further support previous knowledge that a weakened bone-implant interface in osteoporotic bone may not only impede initial stabilization but also increase the risk of implant loosening over time in TSA [19].

With respect to patient outcomes, this study may also support recent literature. In a prospective study with 3577 patients over a period of 20 years, the authors demonstrated that serum calcium phosphate product levels on admission may be a good predictor of postoperative mortality in patients who sustained a proximal femur fracture [20]. In a separate prospective cohort study of 2333 older adults with hip fractures, Li et al. noted that preoperative serum calcium concentrations and postoperative mortality up to an average of 37.5 months follow-up [21]. While these studies comment only on mortality and not on implant-related outcomes, the overall increased complication rate in the setting of elective TSA supplements these findings.

After propensity matching, most of these differences normalized. Two outcomes continued to demonstrate a statistically significant difference: emergency department visits within two months postoperatively (OR 1.4, p < 0.01) and joint loosening/dislocation up to two years postoperatively (OR 1.3, p = 0.03). No other outcomes significantly differed at any of the following time points following propensity match.

While newer studies started to analyze preoperative serum calcium levels as a predictor following geriatric hip fractures, little data exists for implant-related complications [20,21]. For example, in a prospective cohort study, Li et al. report a nonlinear U-shaped association between preoperative serum calcium levels and all-cause mortality in geriatric hip fractures after accounting for confounding variables matching [21]. While the current study is not equipped to identify a U-shaped distribution, given no analysis for hypercalcemic patients, the study does highlight an overall similar rate of non-mortality complications between hypocalcemic and normocalcemic patients after propensity match. More research needs to be done to better understand these relationships, but the data does highlight potential benefit for preoperative optimization of calcium homeostasis prior to elective TSA.

Limitations

Given the database nature of the study, direct conclusions cannot be made about the findings, and, because TriNetX functions as a closed platform with preset regression models and broad windows for laboratory value timings, this limits the study’s reproducibility and ability to perform sensitivity analyses. Secondly, the database used operates by directly pulling CPT and ICD10 codes from the electronic health records of contributing hospitals. Due to this, there is the chance that CPT codes and ICD codes are entered in error and may not correctly reflect each patient. For example, reliance on CPT coding within the TriNetX database prevents this analysis from distinguishing between aTSA and rTSA, elective and trauma-related cases, revision surgeries, and bilateral TSAs. Moreover, precise dates regarding lab values are unavailable within the database, limiting measurements of calcium to broader preoperative windows. Thirdly, the propensity match shows that hypocalcemia must be understood in the context of the underlying medical comorbidities that can contribute to the value. For example, factors like obesity, malnutrition, and smoking could have also been responsible for the increased rates of intraoperative fracture and acute post-operative hematoma/seroma observed before propensity-score matching. Finally, it is possible that low calcium levels observed in the hypocalcemia cohort may have been due to hypoalbuminemia, leading to potential “pseudohypocalcemia” characterized by decreased total calcium but normal ionized calcium [22]. Regardless, despite the aforementioned limitations, the large sample size strengthens the findings reported. Given the potential benefit and relative ease of optimizing calcium homeostasis prior to surgery, the data reported support the need for prospective studies to better understand these findings.

## Conclusions

Prior to propensity score matching, patients with hypocalcemia demonstrated increased odds of implant-related and non-implant-related complications at all timepoints evaluated; however, most complications normalized after propensity score matching. These findings suggest that preoperative hypocalcemia may contribute to select postoperative complications and highlights the potential importance of preoperative metabolic optimization. Future studies are warranted to determine whether correction of hypocalcemia prior to TSA improves postoperative outcomes and reduces complication rates.

## Appendices

**Table 9:** Outcomes of interest and codes utilized. ICD: International Classification of Diseases; CPT: Current Procedural Terminology

Outcome	Codes Utilized
Emergency department visit	1013711 (CPT)
Hospital inpatient admission or observation	1013699 (CPT)
Postoperative hematoma/seroma	M96.83, M96.84 (ICD)
Wound dehiscence	T81.30-32, 998.3 (ICD)
Intraoperative fracture	M96.62 (ICD)
Mechanical loosening or dislocation	T84.028, T84.029, T84.038, T84.039 (ICD)
Prosthetic joint infection	T84.50, T84.59 (ICD)
Arthrotomy or removal of loose body	23105, 23107 (CPT)
Removal of prosthesis	23334, 23335 (CPT)
Revision of prosthesis	23473, 23474 (CPT)
Insertion of drug-delivery implant	11981 (CPT)
Arthroscopy, synovectomy, lysis of adhesions, or debridement	29820-29823, 29825 (CPT)
Incision and drainage of deep abscess or hematoma	23030 (CPT)
Arthrotomy with foreign body removal	23040 (CPT)
